# In Search of Enzymes with a Role in 3′, 5′-Cyclic Guanosine Monophosphate Metabolism in Plants

**DOI:** 10.3389/fpls.2016.00576

**Published:** 2016-05-06

**Authors:** Inonge Gross, Jörg Durner

**Affiliations:** ^1^Nitric Oxide Production and Signalling Group, Institute of Biochemical Plant Pathology, Helmholtz Center MunichGermany; ^2^Chair of Biochemical Plant Pathology, Technische Universität München, FreisingGermany

**Keywords:** nitric oxide, cGMP, phosphodiesterases, plants, guanylate cyclase, signaling

## Abstract

In plants, nitric oxide (NO)-mediated 3′, 5′-cyclic guanosine monophosphate (cGMP) synthesis plays an important role during pathogenic stress response, stomata closure upon osmotic stress, the development of adventitious roots and transcript regulation. The NO-cGMP dependent pathway is well characterized in mammals. The binding of NO to soluble guanylate cyclase enzymes (GCs) initiates the synthesis of cGMP from guanosine triphosphate. The produced cGMP alters various cellular responses, such as the function of protein kinase activity, cyclic nucleotide gated ion channels and cGMP-regulated phosphodiesterases. The signal generated by the second messenger is terminated by 3′, 5′-cyclic nucleotide phosphodiesterase (PDEs) enzymes that hydrolyze cGMP to a non-cyclic 5′-guanosine monophosphate. To date, no homologues of mammalian cGMP-synthesizing and degrading enzymes have been found in higher plants. In the last decade, six receptor proteins from *Arabidopsis thaliana* have been reported to have guanylate cyclase activity *in vitro*. Of the six receptors, one was shown to be a NO dependent guanylate cyclase enzyme (NOGC1). However, the role of these proteins *in planta* remains to be elucidated. Enzymes involved in the degradation of cGMP remain elusive, albeit, PDE activity has been detected in crude protein extracts from various plants. Additionally, several research groups have partially purified and characterized PDE enzymatic activity from crude protein extracts. In this review, we focus on presenting advances toward the identification of enzymes involved in the cGMP metabolism pathway in higher plants.

## Introduction

The second messenger 3′, 5′-cyclic guanosine monophosphate (cGMP) discovered in the 1960s is found in both prokaryotes and eukaryotes (Ashman et al., 1963; Eckstein, 1988; Müller, 1997; Cadoret et al., 2005). The molecule cGMP is synthesized from guanosine triphosphate (GTP) by guanylate cyclase enzymes (GCs) and is involved in various cellular responses, such as protein kinase activity, cyclic nucleotide gated ion channels and cGMP regulated cyclic nucleotide phosphodiesterases (Denninger and Marletta, 1999; Gomelsky and Galperin, 2013). The signal generated by the second messenger is halted by 3′, 5′-cyclic nucleotide phosphodiesterase (PDE) enzymes (Hoyer et al., 1994; Richter, 2002; Maurice et al., 2014). In mammals, the metabolism and physiological role of cGMP is well characterized, this cyclic nucleotide is important in olfactory signaling (Pietrobon et al., 2011), visual adaptation (Vielma et al., 2011) and vasodilation (Thoonen et al., 2013). In contrast, the role of cGMP in plants is not well understood.

**FIGURE 1:**
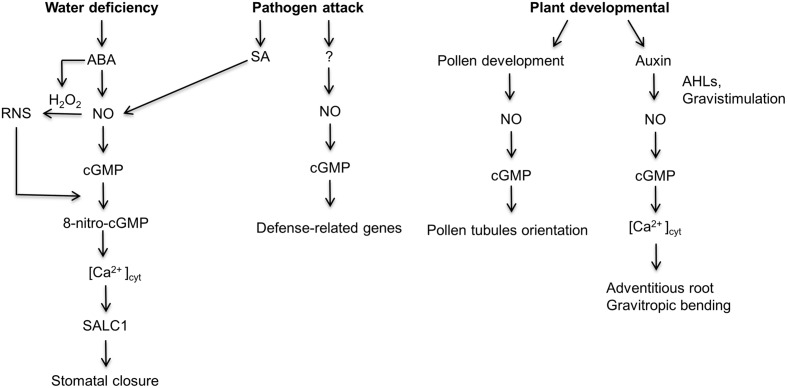
**A schematic depiction of the nitric oxide-induced cGMP signaling pathway in developmental, abiotic and biotic stress processes.** During water stress, an increase in the hormone abscisic acid (ABA) activates the synthesis of nitric oxide (NO). Subsequently, NO stimulates the NO-dependent guanylate cyclase to produce cGMP (Dubovskaya et al., 2011; Joudoi et al., 2013). Concurrently, ABA also activates the production of H_2_O_2_ which reacts with NO to produces reactive nitrogen species (RNS; Joudoi et al., 2013). A reaction between the RNS and cGMP produce 8-Nitro-cGMP which in turn activates the accumulation of cytoplasmic calcium, [Ca2^+^]_cyt_ and the SLOW ANION CHANNEL 1 (SLAC1) which results in stomatal closure. Similarly, during a pathogenic attack, plants close their stomata; however, the NO-cGMP pathway is initiated by the hormone salicylic acid (SA; Hao et al., 2010). Furthermore, during a pathogenic attack, NO-cGMP signaling cascade activates the transcription of the pathogenic marker, PAL in an SA-independent manner (Durner et al., 1998). Furthermore, NO-cGMP signaling pathway is important during pollen tubule development. NO-cGMP signaling pathway is also involved in adventitious root formation stimulated by exogenous and endogenous chemicals, for example, *N*-Acyl-homoserine-lactones (AHLs) produced by gram negative rizobacteria. AHLs promote polar auxin transport which activates the NO-cGMP dependent signaling cascade leading to the development of adventitious root formation (Pagnussat et al., 2003a; Lanteri et al., 2006; Bai et al., 2012). Similarly, gravitropism bending requires the auxin induced NO-cGMP signaling pathway (Hu et al., 2005).

Cyclic nucleotide monophosphates (cNMP) levels including cGMP are lower in plants relative to other eukaryotes and as a result the detection and quantification of cNMP was challenging for several decades (Newton and Smith, 2004). However, with the development of sensitive methods, it is now possible to measure and quantify cNMP *in planta*. The methods include; mass spectrometry based measurements (Newton and Smith, 2004), radiolabeled (Steiner et al., 1972) and antibody (Lomovatskaya et al., 2011) based immunoassays. In addition, non-invasive techniques have been developed that allow the detection of endogenous cytoplasmic cGMP levels *in vivo*, these include a fluorescent cGMP biosensor called FlincG (Isner and Maathuis, 2011) and a cGMP responsive promoter fused to a luciferase reporter gene (Wheeler et al., 2013). Consequently, in the past decade, several research groups have shown a positive correlation between the accumulation of cGMP *in planta* and various developmental processes as well as the response to abiotic and pathogenic stress (**Figure 1**; Durner et al., 1998; Pagnussat et al., 2003b; Szmidt-Jaworska et al., 2004; Maathuis, 2006; Suita et al., 2009; Teng et al., 2010; Joudoi et al., 2013; Li et al., 2014; Nan et al., 2014). For instance, Durner et al. (1998) were the first group to show a correlation between nitric oxide (NO) dependent cGMP synthesis and pathogen defense response in *Nicotiana tabacum*. The elevated levels of cGMP in turn activated the expression of a pathogenic marker, phenylalanine ammonia lyase (PAL). In the subsequent years, several research groups have documented that both NO-dependent and NO-independent cGMP signaling pathways are important in the activation of defense responses during biotic stress (Klessig et al., 2000; Ma et al., 2007; Meier et al., 2009; Pasqualini et al., 2009; Qi et al., 2010; Ma et al., 2013). Additionally, NO-cGMP dependent signaling pathway has been reported to be involved in the development of adventitious roots (Pagnussat et al., 2003a,b; Xuan et al., 2012), stomata closure during abiotic and biotic stress (Neill et al., 2008; Joudoi et al., 2013), protein phosphorylation (Isner et al., 2012; Marondedze et al., 2015) and transcription regulation (Suita et al., 2009).

Collectively, research in the last two decades suggests that cGMP is an important second messenger in plants, albeit, the metabolism of cGMP in plants is not well understood. In this review, we focus on advances toward the identification of enzymes involved in the metabolism of cGMP in plants.

## Nitric Oxide Dependent Guanylate Cyclase Enzymes in Plants

In the plant kingdom, genes coding for nucleotide cyclase (NCs) enzymes have been identified in lower plants from the division of Chlorophyta. For instance *Chlamydomonas reinhardtii* contains more than 90 NCs enzymes (Meier et al., 2007; Marondedze et al., 2016). Among these annotated NCs, NO-induced GC enzymes homologous to those found in mammalian species have been identified (Winger et al., 2008; de Montaigu et al., 2010). In higher plants, protein sequences with high homology to known GCs have not been identified. However, motif searches based on functionally assigned amino acid residues within the catalytic center has resulted in the identification of several proteins that have been shown to have guanylate cyclase activity *in vitro*. These include; phytosulfokine (PSK) receptor, AtPepR1, *Arabidopsis thaliana* guanylate cyclase 1 (AtGC1), brassinosteroid receptor (AtBRII), plant natriuretic peptide receptor (AtPNP-R1) and wall associated kinase-like 10 (AtWAKL10: Ludidi and Gehring, 2003; Kwezi et al., 2007; Meier et al., 2010; Qi et al., 2010; Kwezi et al., 2011; Turek and Gehring, 2016). These receptors synthesize cGMP from GTP independent of NO.

In an effort to identify NO-dependent GCs in plants, Mulaudzi et al. (2011) searched the *Arabidopsis thaliana* sequence database for the conserved residues within the catalytic center as well as the heme-nitric oxide and oxygen binding domain (H-NOX), a domain required for the binding of NO in GCs. The authors found one protein annotated as a Flavin-dependent monooxygenase (At1g62580) that contained both the H-NOX motif and the conserved amino acid residues within the catalytic motif. The enzyme was termed NO dependent guanylate cyclase 1 (NOGC1). Interestingly, stomata closure did not occur in *nogc1* T-DNA knockout mutants lines when treated with an NO donor, 1-hydroxy-2-oxo-3-(3-aminopropyl)-3-isopropyl-1-triazene, compared to wild type plants (NOC5; Joudoi et al., 2013). These exciting results suggest that NOGC1 is involved in the NO-cGMP signaling pathway in regard to stomatal closure. Furthermore, *in vitro* assays confirmed that the recombinant protein NOGC1 has a higher affinity for NO than oxygen (Mulaudzi et al., 2011). However, although NOGC1 recombinant protein is able to synthesize cGMP in an NO dependent manner, cGMP is produced in extremely low amounts (400–450 fmol/μg in 20 min) relative to GCs found in mammals, for example the recombinant sGC from human (940 pmol/min/μg; Kosarikov et al., 2001). It could be possible that additional unknown cofactors are required for the optimal function of this enzyme and therefore, the assay conditions *in vitro* are not ideal. Indeed this is true for the phytosulfokine receptor 1 (PSKR1) which showed an increase in GC activity in the presence of calcium (Muleya et al., 2014). Furthermore, there are additional factors that affect the production of active recombinant proteins; these are discussed in detail by Bernaudat et al. (2011) and references therein. As suggested by Wong and Gehring (2013), it is important that further studies are carried out *in vivo*. For example, the synthesis of cGMP by NOGC1 could be studied in plants containing the cGMP biosensor FlincG (Isner and Maathuis, 2011). The advantage of this system is that the detection of cGMP is non-invasive; therefore, continuous real-time changes in cGMP could be studied. A further advantage is that cytosolic cGMP levels can be detected in specific organelles, thus, allowing high resolution measurements.

In contrast to the recent developments in the identification of NO induced GCs in plants, the publications concerning the identification of plant specific 3′, 5′-cyclic nucleotide phosphodiesterase enzymes (PDEs) responsible for the degradation of cGMP stopped in 2001. It is peculiar that there has been no research output in regard to the identification of PDEs from plants in the last 16 years. The next section provides a summary on the efforts in the identification of PDEs and the comparison of their physical and chemical properties to the PDEs found in prokaryotes, lower and higher eukaryotes.

## 3′, 5′-Cyclic Uanosine Monophosphate Phosphodiesterases Found in Prokaryotic and Eukaryotic Organisms

**FIGURE 2:**
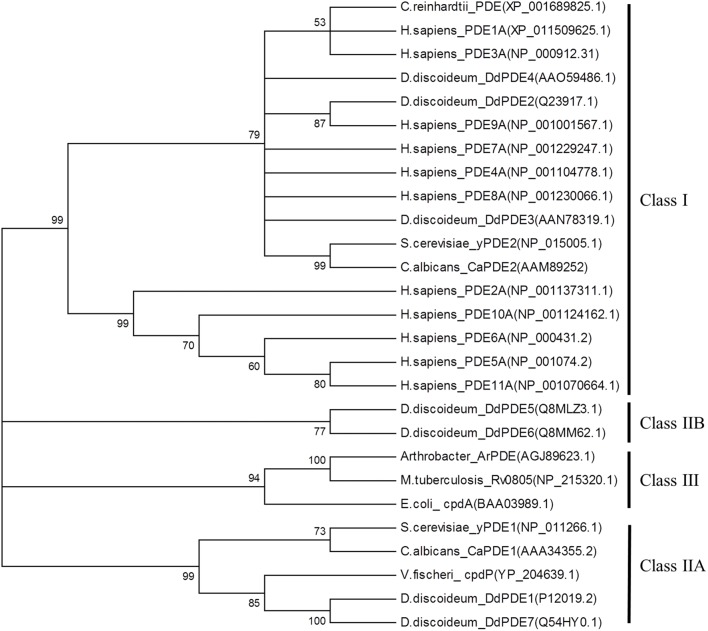
**Phylogenetic analysis of biochemically characterized 3′, 5′-cyclic nucleotide monophosphate phosphodiesterase (PDEs) found in prokaryotes, lower and higher eukaryotes.** The protein sequences of characterized PDEs from Class I, II, and III were retrieved from GenBank. The PDEs belong to *Chlamydomonas reinhardtii* (algae), *Homo sapiens* (human), *Dictyostelium discoideum* (Slime mold), *Saccharomyces cerevisiae* (fungi), *Candida Albicans* (fungi), Arthrobacter (proteobacteria), *Escherichia coli* (proteobacteria), *Mycobacterium tuberculosis* and *Vibrio fischeri*. A multiple alignment with the retrieved PDEs was performed in a protein alignment program, Muscle (Edgar, 2004). The phylogenetic tree was constructed in MEGA6 (Tamura et al., 2013) using the Neighbor-joining statistical method with a bootstrap replication number of 1000. Members of the Class I PDEs are found in lower and higher eukaryotes and Class II PDEs are found in lower eukaryotes as well as bacteria. Finally, Class III PDEs are found exclusively in bacteria.

3′, 5′-cyclic nucleotide phosphodiesterase enzymes (PDEs) degrade cGMP and the other second messenger, 3′, 5′-cyclic adenosine monophosphate (cAMP). The PDE superfamily is classified into three groups (Class I, Class II, and Class III) based on sequence and structural similarities (**Figure 2**). Class I PDEs are found in higher and lower eukaryotic organisms including all mammalian species and numerous species belonging to the Amoebozoa and Fungi kingdoms (Sass et al., 1986; Michaeli et al., 1993; Rosman et al., 1997; Thomason et al., 1998; Fawcett et al., 2000; Fujishige et al., 2000; Hetman et al., 2000; Kuwayama et al., 2001; Wang et al., 2001; Lee et al., 2002; Richter, 2002; Huai et al., 2003; Jung and Stateva, 2003; Wang et al., 2003; Zhang et al., 2005; Bader et al., 2006; Corbin et al., 2006; Muradov et al., 2010; DeNinno et al., 2011; Barnes, 2013; Zhu et al., 2013; Du et al., 2014; Maurice et al., 2014). Class II PDEs are found in lower eukaryotes and bacteria (Van Haastert et al., 1983; Van Haastert and Van Lookeren Campagne, 1984; Lacombe et al., 1986; Dunlap and Callahan, 1993; Hoyer et al., 1994; Imamura et al., 1996; Degerman et al., 1997; Bosgraaf et al., 2002; Bader et al., 2007; Tian et al., 2014). Finally, members of class III belong exclusively to PDEs isolated from prokaryotes (Imamura et al., 1996; Richter, 2002; Shenoy et al., 2005; Zheng et al., 2013). Although Class I, II, and III PDEs have different sequence and structure homology, they use similar mechanisms in the hydrolysis of cNMP by cleaving the ester bond at carbon 3′ to produce a non-cyclic 5′ NMP. These enzymes belong to a superfamily called binuclear metallohydrolases (Mitić et al., 2006).

### Partially Purified PDEs from Plants with Promiscuous Enzyme Activity

In the plant kingdom, lower plants from the division of Chlorophyta contain genes coding for classic Class I PDEs within their genome. As shown by our phylogenetic tree analysis, a PDE from *Chlamydomonas reinhardtii* is closely related to mammalian Class I PDE 1A and 3A (**Figure 2**). These findings complement studies by Fischer and Amrhein (1974) who showed that a partially purified protein from *C. reinhardtii* exhibited typical Class I enzyme activity during the hydrolysis of cAMP and cGMP. This enzyme was also able to hydrolyze 3′, 5′-cyclic cytosine monophosphate (cCMP) and the hydrolysis was threefold and sixfold higher than cAMP and cGMP, respectively. These findings are compatible with recent data that shows that mammalian PDEs exhibit promiscuous substrate specificity toward other 3′, 5′-cyclic nucleotides than previously reported, for example, cyclic uridine monophosphate (cUMP), cyclic thymidine monophosphate (cTMP) and cyclic inosine monophosphate (cIMP; Reinecke et al., 2011).

In higher plants such as angiosperms, PDEs have not been identified; however, PDE activity has been detected in crude protein extracts from various plants. In an effort to purify PDEs, crude extract from *Solanum tuberosum* (Ashton and Polya, 1975; Zan-Kowalczewsk et al., 1984), *Pisum sativum* (Lin and Varner, 1972), *Nicotiana tabacum* (Shinshi et al., 1976; Matsuzaki and Hashimoto, 1981) and *Solanum lycopersicum* (Abel et al., 2000) were subjected to sequential purification steps that included ammonium sulfate precipitation, ion chromatography and gel filtration chromatography. Molecular weights of protein fractions with PDE activity were determined by gel filtration with markers of known molecular weight. Proteins capable of hydrolyzing cNMP were present as monomeric as well as tetrameric protein complexes. The molecular weight values for the monomer and tetramer PDEs range from 60 000–75 000 and 270 000–350 000, respectively and they all possess similar enzymatic activity properties. In other plants, the partially purified PDEs appear in fractions at lower molecular weights than the proposed size of the monomeric form, 60 000–75 000. In *Spinacea oleracea*, the sizes of partially purified PDEs with estimated sizes of 50 and 37 KDa were reported (Brown et al., 1979). All partially purified PDEs mentioned were from crude cytosolic (soluble) proteins. However, Vandepeute et al. (1973) report the partial purification of PDE from both soluble proteins and particulate (membrane) proteins, suggesting that plants could possess both cytosolic and membrane bound PDEs.

In contrast to Class I, II, and III PDEs, all mentioned PDEs above hydrolyze 2′, 3′ and 3′, 5′-cNMPs with most of them showing a higher affinity toward the hydrolysis of 2′, 3′ cNMP than 3′, 5′-cNMP (Supplementary Table S1; Lin and Varner, 1972; Ashton and Polya, 1975; Brown et al., 1979; Endress, 1979; Abel et al., 2000). It is probable that unlike other organisms, plants contain PDEs with dual enzymatic function. This claim is supported by the finding from a partially purified PDE (70 kDa) from *S. lycopersicum* that was shown to be induced and excreted from suspension-cultured cells during inorganic phosphate deficiency (Abel et al., 2000). It was proposed that together with ribonuclease I and acid phosphatases, plant PDEs are expressed and secreted to degrade extracellular RNA, releasing inorganic phosphate that is in turn transported into the cells via Pi transporters. The authors speculate that due to its higher affinity for 2′, 3′ cNMPs than 3′, 5′-cNMPs and because it is excreted during phosphate deficiency, the PDE identified is involved in RNA turnover rather than the degradation of 3′, 5-cNMP and thus the enzyme was named 2′, 3′-cyclic nucleotide-phosphodiesterase. An alternative suggestion is that in plants, PDEs and nucleotide pyrophosphates are part of a multi-complex structure and because they possess similar properties such as isoelectric point and molecular weight, they co-purify during the isolation process. This is a more convincing theory as other groups have reported partially purified PDEs that specifically degrade 3′, 5′-cNMP isomers; these are discussed in the following section.

### Partial Purification of 3′, 5′-Cyclic Nucleotide Monophosphate Specific PDEs in Plants

To isolate protein with PDE activity from *Phaseolus vulgaris*, the authors extracted total soluble extract from the vegetative tissue and centrifuged it at 100 000 *g* (Brown et al., 1977). The pellet was retained and dissolved in 50 mM Tris–HCL buffer (pH7.4) and the following purification steps were performed; ammonium sulfate precipitation and gel filtration (Sephadex G-200). Two fractions containing PDE activity were detected with molecular weights of 340 and 76 KDa, confirming that in plants PDEs could exist in monomeric and tetrameric forms. In addition, both enzyme fractions produced a mixture of 3′ NMP and 5′ NMP after the hydrolysis of 3′, 5′-cNMPs, similar to all partially purified plant PDEs described to date. Interestingly, PDE from *P. vulgaris* was described to specifically hydrolyze 3′, 5′-cNMP and had no activity against 2′, 3-cAMP. The substrate preference is in the following order: 3′, 5′-cAMP, 3′, 5′-cGMP, 3′, 5′-cUMP and 3′, 5′-cCMP (Brown et al., 1977). Similarly, partially purified PDE from *S. oleracea* and *P. volganic* were reported to have catalytic preference for 3′, 5′-cNMP (Brown et al., 1980; Diﬄey et al., 2001). The PDE from *S. oleracea* hydrolyzed both 3′, 5′-cAMP and 3′, 5′-cAMP and divalent metals were not required for optimal enzyme activity. The hydrolysis of 3′, 5′-cAMP and 3′, 5′-cAMP by the partially purified PDE from *P. volganic* was activated and inhibited by Mg^2+/^Mn^2+^ and Fe^3+^, respectively. These results indicate that the PDE from *P. volganic* requires divalent metals similar to CLASS I PDEs found in eukaryotic organisms. However, in contrast to CLASS I PDEs, the presence of Fe^3+^ activated the hydrolysis of 3′, 5′-cCMP (Diﬄey et al., 2001).

### The Future for Plant Specific PDE Research

It is possible to speculate that with the advances in proteomics in the last 16 years, there is a high chance that plant-specific PDEs could be identified from the sequentially fractionated samples. For example, the refined liquid chromatography coupled mass spectrometry (LC MS/MS) based proteomics analysis techniques are now capable of deciphering protein mixtures. The sensitivity of the techniques allows the identification of proteins in low abundance (Ahlf et al., 2013; Di Girolamo et al., 2013; Fukao et al., 2013). Furthermore, peptides fingerprints/proteins from the mass spectrometry analysis can be readily identified as the genomes of 49 plants species have been sequenced since 2001 (Michael and Jackson, 2013). These include the genomes of *S. tuberosum* (Xu et al., 2011), *N. tabacum* (Sierro et al., 2014), and *S. lycopersicum* (Daniell et al., 2006).

## Conclusion

Nitric oxide-dependent cGMP production is involved in various signaling processes in plants particularly in (i) the control of stomatal aperture which is important in surviving water deficit (Neill et al., 2008; Joudoi et al., 2013) and (ii) defense response during pathogen attack (Durner et al., 1998; Ma et al., 2013). NOGC1 is the first NO-dependent GC identified in plants; however, the function of this protein remains to be elucidated *in planta*. As stated, the last publication on identifying plant specific PDEs was in 2001. Finding plant PDEs could have a substantial impact in understanding the NO-cGMP pathway and its physiological effects. As cGMP is positively correlated with plants adapting to environmental stresses, the discovery of PDEs will lead to the development of plant-specific PDE inhibitors which would maintain intracellular levels of cGMP. In mammals, perturbation in the synthesis as well as degradation of cGMP results in various human ailments (Maurice et al., 2014). Inhibitors of mammalian PDEs are used as therapeutic agents to regulate the concentrations of intracellular cGMP and thus alleviate diseases. It is safe to postulate that plant specific PDE inhibitors could be important in improving crop performance during environmental stress and thus could have similar commercial value as the well-studied mammalian PDEs.

## Author Contrbutiions

The first and corresponding author, IG initiated and wrote the review. JD proof-read the manuscript and gave intellectual input.

## Conflict of Interest Statement

The authors declare that the research was conducted in the absence of any commercial or financial relationships that could be construed as a potential conflict of interest.
