# Supplementary material for: In Search of Enzymes with a Role in 3′, 5′-Cyclic Guanosine Monophosphate Metabolism in Plants
Source: Front Plant Sci. 2016 May 6;7:576. doi: 10.3389/fpls.2016.00576 (PMC4858519; doi:10.3389/fpls.2016.00576)
Supplement: Supplementary file 1 [file Table_1.DOC]

Supplementary Table 1. A summary of physical and enzymatic properties of 3’5-cyclic nucleotide phosphodiesterase found in prokaryotic and eukaryotic organisms

| **Kingdom** | **Name** | **Class** | **Accession** | **Size (kD)** | **Co-factors** | **Inhibitors** | **Origin** | **Hydrolysis** | **Reference** |
| --- | --- | --- | --- | --- | --- | --- | --- | --- | --- |
| **Fungi** |  |  |  |  |  |  |  |  |  |
|  | yPDE 1 | II | NP_015005.1 | 60 | None | DTT | *Saccharomyces cerevisiae* | 3’, 5’-cAMP 3’, 5’cGMP | Tian et al., 2014 |
|  | yPDE 2 | I | NP_011266.1 | 42 | N/A | N/A | *Saccharomyces cerevisiae* | 3’, 5’-cAMP | SASS et al., 1986 |
|  | CaPDE 1 | II | AAA34355.2 | 47 | None | Cu2+, Zn2+, DTT | *Candida albicans* | 3’, 5’-cAMP 3’, 5’cGMP | Hoyer et al., 1994 |
|  | CaPDE 2 | I | AAM89252.1 | 65 | N/A | N/A | *Candida albicans* | 3’, 5’-cAMP | Jung and Stateva, 2003 |
| **Bacteria** |  |  |  |  |  |  |  |  |  |
| Actinobacteria | ArPDE | III | AGJ89623.1 | 34 | Co^2+^, Mn^2+^ | Zn^2+^, NaF | *Arthrobacter* | 3’, 5’-cAMP 3’, 5’cGMP | Zeng et al., 2013 |
|  | Rv0805 | III | NP_215320.1 | 34.2 | Mg^2+^,Mn^2+^ | N/A | *mycobacterium tuberculosis* | 3’, 5’-cAMP 3’, 5’cGMP | Shenoy et al., 2005 |
| Proteobacteria | cpdA | III | BAA03989.1 | 30.9 | Fe^2+^ | N/A | *Escherichia coli* | 3’, 5’-cAMP | Imamura et al., 1996 |
|  | cpdP | II | YP_204639 | 36 | N/A | N/A | *Vibrio fischeri* | 3’, 5’-cAMP, | Dunlap and Callahan, 1993 |
| **Mammals** |  |  |  |  |  |  |  |  |  |
|  | PDE 1 | I | XP_011509625.1 | 64.8 | CaM, Ca^2+^ | IBMX, Vinpocetine,  8-methoxyIMBX | *Homo sapiens* | 3’, 5’-cAMP, 3’, 5’cGMP | Synder et al., 1999 |
|  | PDE 2 | I | NP_001137311.1 | 105 | Mg^2+^ | EHNA, BAY60-7550 | *Homo sapiens* | 3’, 5’-cAMP, 3’, 5’cGMP | Rosman et al., 1997  Zhu et al., 2013 |
|  |  |  |  |  |  |  |  |  |  |
|  | PDE 3 | I | NP_000912.31 | 124 | Mg^2+^ | Cilostamide, enoximone, lixazinone, theophylline | *Homo sapiens* | 3’, 5’-cAMP, 3’, 5’cGMP | Degerman et al., 1997, Barnes, 2013 |

| **Kingdom** | **Name** | **Class** | **Accession** | **Size (kD)** | **Co-factors** | **Inhibitors** | **Origin** | **Hydrolysis** | **Reference** |
| --- | --- | --- | --- | --- | --- | --- | --- | --- | --- |
| **Mammals** | PDE 4 | I | NP_001104778.1 | 95.2 | Mg^2+^ | Rolipram, cilmomilast, roflumilast, Zardaverine, theophylline | *Homo sapiens* | 3’, 5’-cAMP | Lee et al., 2002  Huai et al., 2003  Barnes, 2013 |
|  | PDE 5 | I | NP_001074.2 | 100 | Mg^2+^ , Zn^2+^ | DMPPO, IBMX, theophylline, zaprinast, tadalafil, dipyridamole, sildenafil, vardenafil | *Homo sapiens* | 3’, 5’cGMP | Hill et al., 1998  Wang et al., 2001  Corbin et al, 2006  Maurice et al., 2014  Barnes, 2013 |
|  | PDE 6 | I | NP_000431.2 | 99 | Mg^2+^ | Vardenafil, sildenafil, Zaprinast | *Homo sapiens* | 3’, 5’cGMP | Zhang et al., 2005  Muradov et al., 2010 |
|  | PDE 7 | I | NP_001229247.1 | 55 | Mg^2+^ | Theophylline, ASB16165, BRL5081 | *Homo sapiens* | 3’, 5’-cAMP | Michaeli et al., 1993  Maurice et al., 2014  Barnes, 2013 |
|  | PDE 8 | I | NP_001230066.1 | 86 | Mg^2+^ , Mn^2+^, Ca^2+^ | Tetrahydroisoquinoline derivative | *Homo sapiens* | 3’, 5’-cAMP | Fisher et al., 1998; Maurice et al., 2014  DeNinno et al., 2011 |
|  | PDE 9 | I | NP_001001567.1 | 61 | Mg^2+^ , Mn^2+^ | BAY73 6691, theophylline, zaprinast, sildenafil | *Homo sapiens* | 3’, 5’cGMP | Maurice et al., 2014  Barnes, 2013  Wany et al., 2003 |
|  | PDE 10 | I | NP_001124162.1 | 89 | Mg^2+^ | Papaverine, IBMX, SCH51866, E4021, Zaprinast, dipyridamole | *Homo sapiens* | 3’, 5’-cAMP, 3’, 5’cGMP | Fujishige et al., 2000  Maurice et al., 2014 |
|  | PDE 11 | I | NP_001070664.1 | 55 | Mg^2+^ | Papaverine, IBMX, dipyridamole, Zaprinast | *Homo sapiens* | 3’, 5’-cAMP, 3’, 5’cGMP | Fawcett et al., 2000  Hetman et al*.*, 2000  Maurice et al., 2014 |

| **Kingdom** | **Name** | **Class** | **Accession** | **Size (kD)** | **Co-factors** | **Inhibitors** | **Origin** | **Hydrolysis** | **Reference** |
| --- | --- | --- | --- | --- | --- | --- | --- | --- | --- |
| **Amoebozoa** |  |  |  |  |  |  |  |  |  |
|  | DdPDE1(PsdA) | II | P12019.2 | 51 | N/A | DTT | *Dictyostelium discoideum* | 3’, 5’-cAMP  3’, 5’cGMP | Lacombe et al., 1986  Van Haastert et al., 1983,  Bader et al., 2007 |
|  | DdPDE2 (regA) | I | Q23917.1 | 91 | Mg^2+^ | Trequinsin, dipyridamole, MY-5445, Etazolate | *Dictyostelium discoideum* | 3’, 5’-cAMP | Du et al., 2014  Thomason et al., 1998 |
|  | DdPDE3 | I | AAN78319.1 | 60 | Mg^2+^ , Mn^2+^ | IBMX | *Dictyostelium discoideum* | 3’, 5’cGMP | Kuwayama et al., 2001 |
|  | DdPDE4 | I | AAO59486.1 | 89 | Mg^2+^ , Mn^2+^ | IBMX | *Dictyostelium discoideum* | 3’, 5’-cAMP | Bader et al., 2006 |
|  | DdPDE5(GbpA) | II | Q8MLZ3.1 | 98 | Mg^2+^ | N/A | *Dictyostelium discoideum* | 3’, 5’cGMP | Bosgraaf et al., 2002  Van Haastert and Van Lookeren, 1984 |
|  | DdPDE6(GbpB) | II | Q8MM62.1 | 125 | Mg^2+^ , | N/A | *Dictyostelium discoideum* | 3’, 5’-cAMP, 3’, 5’cGMP | Bosgraaf et al., 2002 |
|  | DdPDE7 | II | Q54HY0.1 | 48 | N/A | DTT | *Dictyostelium discoideum* | 3’, 5’-cAMP, 3’, 5’cGMP | Bader et al., 2007 |
